# Implementation of modified Seldinger technology for percutaneous catheterization in critically ill newborns

**DOI:** 10.1590/1980-220X-REEUSP-2022-0347en

**Published:** 2023-06-30

**Authors:** Higor Pacheco Pereira, Izabela Linha Secco, Andrea Moreira Arrué, Letícia Pontes, Mitzy Tannia Reichembach Danski

**Affiliations:** 1 Universidade Federal do Paraná, Hospital Infantil Waldemar Monastier, Campo Largo, PR, Brazil.; 2 Instituto Federal do Paraná, Palmas, PR, Brazil.; 3 Universidade Federal do Paraná, Departamento de Enfermagem, Curitiba, PR, Brazil.

**Keywords:** Newborn, Catheterization, Peripheral, Intensive Care Units, Neonatal, Technology, Evidence-Based Nursing, Recién Nacido, Cateterismo Periférico, Unidades de Cuidados Intensivos Neonatales, Tecnología, Enfermería Basada en la Evidencia, Recém-Nascido, Cateterismo Periférico, Unidades de Terapia Intensiva Neonatal, Tecnologia, Enfermagem Baseada em Evidências

## Abstract

**Objective:**

To describe the implementation of Modified Seldinger Technology for percutaneous catheterization in critically ill newborns.

**Method:**

A quasi-experimental before- and-after study, carried out with neonatologist nurses in a Neonatal Intensive Care Unit.

**Results:**

Seven nurses participated in the research. Catheter pre-insertion, insertion and maintenance were assessed using the conventional and modified Seldinger technique. Reliability was satisfactory in pre-test, 5.40 (Md = 6.00), and post-test, 5.94 (Md = 7.00), and perfect in the items about device insertion and maintenance. There was low assertiveness in the items on indication, microintroduction procedure via ultrasound, limb repositioning and disinfection of connections/connectors.

**Conclusion:**

Despite the Modified Seldinger Technique expanding some stages of execution over the traditional method of percutaneous catheterization, nurses were more assertive after theoretical-practical training. The technology was implemented and is in the process of being implemented in the health service.

## RESUMO

**Objetivo:**

Descrever a implantação da Tecnologia de Seldinger Modificada para cateterismo percutâneo em recém-nascidos criticamente enfermos.

**Método:**

Estudo quase-experimental do tipo antes e depois, realizado com enfermeiros neonatologistas em Unidade de Terapia Intensiva Neonatal.

**Resultados:**

Sete enfermeiros participaram da pesquisa. Avaliou-se pré-inserção, inserção e manutenção do cateter por meio da técnica convencional e de Seldinger modificada. A confiabilidade foi satisfatória no pré-teste, 5,40 (M_d_= 6,00), e pós-teste, 5,94 (M_d_= 7,00), e perfeita nos itens sobre inserção e manutenção do dispositivo. Houve baixa assertividade nos itens sobre indicação, procedimento de microintrodução via ultrassom, reposicionamento do membro e desinfecção das conexões/conectores.

**Conclusão:**

Apesar de a Técnica de Seldinger Modificada ampliar algumas etapas de execução sobre o método tradicional de cateterismo percutâneo, os enfermeiros obtiveram maior assertividade após capacitação teórico-prática. A tecnologia foi implantada e encontra-se em processo de implementação no serviço de saúde.

## RESUMEN

**Objetivo:**

Describir la implementación de la Tecnología Seldinger Modificada para el cateterismo percutáneo en recién nacidos en estado crítico.

**Método:**

Estudio cuasi-experimental antes y después, realizado con enfermeras neonatólogas en una Unidad de Cuidados Intensivos Neonatales.

**Resultados:**

Siete enfermeros participaron de la investigación. La preinserción, inserción y mantenimiento del catéter se evaluaron mediante la técnica de Seldinger convencional y modificada. La fiabilidad fue satisfactoria en la preprueba 5,40 (M_d_= 6,00) y posprueba 5,94 (M_d_= 7,00) y perfecta en los ítems de inserción y mantenimiento del dispositivo. Hubo baja asertividad en los ítems sobre indicación, procedimiento de microintroducción por ultrasonido, reposicionamiento de extremidades y desinfección de conexiones/conectores.

**Conclusión:**

A pesar de que la Técnica de Seldinger Modificada amplió algunas etapas de ejecución sobre el método tradicional de cateterismo percutáneo, los enfermeros se mostraron más asertivos después del entrenamiento teórico-práctico. La tecnología fue implementada y está en proceso de implementación en el servicio de salud.

## INTRODUCTION

Hospitalized newborns (NB) often require prolonged venous access for drug administration and parenteral nutrition. According to international recommendation^(1)^, peripheral catheters are indicated for this clientele if intravenous therapy is prescribed for up to seven days and if the drug allows administration via a peripheral route. Therefore, it is concluded that peripheral devices are unfeasible for NB, due to the need for solutions that require long-term central administration^(2)^. Thus, a central line in critically ill NB becomes mandatory.

The use of a peripherally inserted central catheter (PICC) as an innovative technology in infusion therapy began to be used in Brazil more than three decades ago, when nurses became the professionals most involved in its execution^(3)^. PICC use prevailed over other traditional catheters for many reasons: lower risk of infection, cost-effective and convenient insertion at the bedside^(4,5)^, reduced patient discomfort due to multiple puncture attempts, resistance to the system peripheral venous hyperosmolarity and preservation^(6)^. Another aspect of fundamental importance in the prevention of complications and iatrogenic events is the fact that this central venous catheter (CVC) has a peripheral insertion, which potentially prevents the occurrence of pneumothorax^(7)^.

However, it is widely recognized that PICC placement in NB presents a unique set of technical challenges, which are even more potent in preterm infants. Therefore, tools that can increase the success rate and reduce complications associated with the procedure are invaluable^(8)^. Faced with this difficulty, some technologies have been developed to improve percutaneous catheterization in NB and provide improvements in the health care of this population. One of them is the Modified Seldinger Technique (MST).

Originally, the Seldinger technique has been around since the 1950s, known as classical Seldinger. From technological refinements, this evolved notably, which resulted in greater security in the insertion of central lines. After these increments, the classic technique was modified to assist patients who demand more delicate care, such as NB, which culminated in the nomenclature of the technology^(9)^.

MST, in turn, adds some steps in relation to the traditional PICC insertion method, as access to the venous network is established through a small-gauge needle, followed by the insertion of a malleable guide wire. After introducing approximately 5 cm of the guide, the needle used for the initial puncture is replaced by a peel-away dilator, which has only the plastic part. Then, the guidewire is withdrawn, and the catheter is introduced through the dilator until the desired measurement is reached. At the end of the progression of the device, the peel-away, which is also bipartite, is broken and the PICC is in situ^(10)^.

Although previous technologies predominate in clinical practice for PICC insertion, it is already recognized that MST has achieved prominence for making central venous catheterization minimally invasive in neonates^(11)^. Moreover, MST complies with the recommendations of guidelines on insertion techniques for central venous catheters and actions aimed at reducing adverse events^(12,13)^.

What makes MST less traumatic is the use of thinner and more malleable introducers and guides. This characteristic also called this technique microintroduction^(13)^, contributing to reduce the risk of thrombosis, infection and bleeding during the procedure^(14)^, in addition to providing a successful and less traumatic experience for NB^(15)^.

For its use to be satisfactory, access to the venous network requires a trained professional and the use of technologies for venous device insertion^(16)^. With regard to the implementation of technologies in patient care, it is necessary to train and qualify the team on which it is intended, with periodic updates in order to improve the ability, in particular, of procedures not performed routinely^(17)^.

Given the above, the aim of this study was to describe the implementation of MST for percutaneous catheterization in critically ill NB.

## METHODS

### Study Design

This is a quasi-experimental before-and-after study. It is noteworthy that the MST was implemented in this study and is in the implementation phase in the health service. This research shows the initial phase of a randomized clinical trial entitled “*Efetividade da Punção com a Técnica de Seldinger Modificada no cateter central de inserção periférica*”, with the Brazilian Registry of Clinical Trials (ReBEC) RBR-69vks36.

### Population

Nurses from the Neonatal Intensive Care Unit (NICU) where the study was developed who provided direct NB care participated. These are professionals qualified in conventional percutaneous catheterization and responsible for the insertion and maintenance of this vascular device.

### Site

Data collection took place in the NICU of a reference pediatric public hospital, specialized in the care of children and adolescents in the southern region of Brazil. This unit is a reference service in the state for highly complex assistance to NB who use the Brazilian Health System (*Sistema Único de Saúde*). It offers 20 beds, human and material resources necessary to provide uninterrupted support to the vital functions of hospitalized NB. The service is made up of 113 professionals, such as nurses, nursing technicians, physicians on duty and physiotherapists who work full time, in addition to psychologists, speech therapists, social workers and occupational therapists.

The use of PICC inserted by the conventional technique in the aforementioned NICU has been taking place since its inauguration in 2009, as it is an indispensable device in assisting this clientele. All PICC insertion procedures are performed at the bedside and any intravenous administration in the unit is performed with the aid of infusion pumps by nursing professionals.

### Selection Criteria

All nurses working at the study NICU who consented to participate in the research were included in the survey. Nurses removed from their duties during the training period were excluded from the study.

### Data Collection

The data collection period took place between June and November 2021. The survey was carried out in three stages, which will be described in detail below. In the first, theoretical and practical training of the research team on the MST technique in neonates was carried out (between January and February 2021). In the second stage, data collection instruments were prepared (pre-/post-test) (from March to May 2021). Finally, in the third stage, study participants received theoretical and practical training on MST technology (from June to November 2021), taught by the duly trained research team.

In the first stage, two nurses specializing in child health were trained by the technology producing industry based on infusion therapy guidelines. Previously scheduled meetings, training on a virtual platform and in person were held in the study setting, with the use of an appropriate mannequin, totaling 30 hours of training in MST. In the second stage, instruments based on national and international recommendations^(1,12,18–21)^ on PICC insertion techniques were prepared by the researchers and subsequently compiled in pre-/post-test.

This instrument had questions that assessed PICC pre-insertion and maintenance, either by conventional technique or MST, and questions referring to PICC insertion by MST. The questionnaire ended with 37 questions, and had “true” or “false” as answer options.

In the third stage, there was the training of nurses, previously scheduled, in after shift work. It was held in the study site’s auditorium, with a total duration of six hours. Initially, participants completed pre-test. Next, theme theorization was carried out through the expository-dialogue method, followed by practical activity, taught by specialist researchers and an experienced nurse in MST.

Nurses performed the puncture procedure with the proposed technology on the mannequin in the hospital’s auditorium, accompanied and guided by researchers and expert nurses until the technique was fully and adequately developed, following the step-by-step instructions shown in theory. If professionals did not perform a step or needed to be reminded, they performed the technique again on the dummy until the correct execution. This monitoring took place through observation with a field diary completed by the research team. At the end of the theoretical-practical training, the post-test instrument was applied with the same questions asked in pre-test, with the aim of verifying whether there was agreement in the answers in the two moments.

### Data Analysis and Treatment

The information was tabulated in an Excel^®^ spreadsheet with independent typing, and analyzed in the Statistical Package for the Social Sciences (SPSS), version 20.0. In descriptive statistics, data were described in absolute and relative frequencies and measure of central tendency (M_e_ = Mean; M_d_ = Median). Pre- and post-test reliability assessment was performed using the Kappa coefficient, which consists of the proportion of agreement in the responses before and after professional training. Values were interpreted according to the Landis & Koch classification. McNemar’s χ^2^ test was used to verify equality between the proportions of responses (pre- and post-test), and values of p ≤ 0.05 were considered significant.

Paired t test was used in related parametric samples (pre- and post-test) by professional category, in order to assess whether the means of the two measures are statistically different. Values of p ≤ 0.05 between the means were considered significant.

### Ethical Aspects

The research complies with the guidelines of Resolution 466/12 of the Brazilian National Health Council, and was approved by the Research Ethics Committee on January 12, 2021, in accordance with favorable opinion 4,495,894, (CAAE (*Certificado de Apresentação para Apreciação* Ética – Certificate of Presentation for Ethical Consideration) 39827120.2.0000.0102). The information records were identified by codes as follows: health professional (HP1, HP2, HP3, HP4…), respecting participants’ anonymity.

## RESULTS

The study scenario has 8 (n = 8) clinical nurses. After applying the eligibility criteria, seven nurses (n = 7) became research participants. One (n = 1) was excluded because he was away from work activities during the research training period.

Regarding the profile of these professionals, all performed care activities in the NICU and were between 34 and 43 years old (Me = 38 years old). Training time ranged from 12–15 years (Me = 13 years), and time working in the unit ranged from one to 12 years (Me = 9 years).

Regarding the training of participants, Table 1 shows the hits in pre- and post-test of nurses on care in PICC pre-insertion in MST.

**Table 1.** Hits in pre- and post-test of nurses participating in the research (n = 7) on peripherally inserted central catheter pre-insertion in the training of the Modified Seldinger Technique – Campo Largo, PR, Brazil, 2021.

Pre-insertion questions	Pre-test hit	Post-test hit	p
1. Once formally qualified through a course and regulated by the Class Council, nurses can insert the PICC, regardless of the technique to be used.	3 (43%)	5 (71%)	0.500* 0.462^†^
2. Nurses must perform a physical examination of patients’ venous network using the inspection and palpation technique, tourniqueting the limb to visualize the vein and using a tape measure to measure the length of the catheter to be introduced.	7 (100%)	7 (100%)	1^†^
3. The catheter gauge must be previously chosen, taking into account NB’s weight and size as well as aspects of the condition of patients’ venous network. The PICC diameter should not exceed one third of the internal diameter of the chosen vein.	7 (100%)	7 (100%)	1^†^
4. It is recommended to indicate PICC after six days of intravenous therapy. Thus, NB who underwent unsuccessful peripheral venipuncture and have a difficult venous network do not meet the PICC insertion criteria and should receive another type of central catheter.	4 (57%)	5 (71%)	1* 0.552^†^
5. In NB with a history of difficult peripheral venous access, PICC insertion should be considered early, in order to have a greater chance of success with the insertion procedure.	7 (100%)	7 (100%)	1^†^
6. In neonatology, the main indications for PICC insertion are intravenous therapy after 6 days and administration of fluids or irritating and/or vesicant medications.	6 (86%)	6 (86%)	1* 0.167^†^
7. There are some contraindications for PICC insertion, such as impaired venous network visualization/identification, thrombocytopenia and coagulation disorders, presence of skin lesions, infection, phlebitis and infiltration close to the planned puncture site.	7 (100%)	7 (100%)	1^†^
8. Guardians’ legal consent of NB for PICC insertion is not required in an emergency.	3 (43%)	3 (43%)	1* 0.417^†^
9. For insertion in lower limbs, position the measuring tape at the site chosen for puncture, run the tape to the inguinal region of the same member, from this point to the umbilicus and then to the xiphoid process.	7 (100%)	7 (100%)	1^†^
10. For insertion in the upper regions (arms, jugular veins and head), position the measuring tape at the site chosen for the puncture and run it towards the right wishbone and then to the second intercostal space.	6 (86%)	7 (100%)	
11. The microintroduction can only be performed with the aid of ultrasound (guided or assisted puncture) so that, without this imaging equipment, it is not possible to perform the technique in its entirety.	3 (43%)	5 (71%)	0.625* 0.077^†^
12. Provide measures for pain control before and during the procedure. Use non-pharmacological measures such as non-nutritive sucking with sucrose or breast milk, restraint and swaddling. Also consider administering opioids 30 minutes before the procedure, such as fentanyl. In addition to mitigating pain, measures are necessary to reduce NB’s psychomotor agitation, ensuring safety and access to the target vessel.	7 (100%)	7 (100%)	1^†^
13. The basilic vein is the first vein of choice for PICC insertion, as it is smaller in length and has fewer valves, ensuring better catheter progression.	7 (100%)	7 (100%)	1^†^
14. The cephalic vein is the second vein of choice. It is more superficial than the basilica, more extensive and presents a slight curve in its most distal portion, which may be a factor that hinders catheter progression.	7 (100%)	7 (100%)	1^†^
15. The brachial vein is the third vein of choice. In this case, we have to select it, ensuring that the brachial artery will not be inadvertently punctured.	7 (100%)	7 (100%)	1^†^
16. Other puncture alternatives in neonatology are the scalp (temporal and auricular), cervical (internal jugular) and lower limbs (great saphenous, popliteal).	2 (28%)	3 (43%)	1.000* 0.696^†^
**Total**	**7 (100%)**	**7 (100%)**	

Source: Survey data, 2021. Note: NUR = applied to nurses. *McNemar’s χ2 test; ^†^Kappa coefficient.

Table 2 describes the number of hits in nurses’ pre- and post-test regarding the necessary care for insertion from the MST.

**Table 2.** Hits in pre- and post-test of nurses participating in the research (n = 7) on peripherally inserted central catheter insertion in the training of the Modified Seldinger Technique – Campo Largo, PR, Brazil, 2021.

Insertion questions	Hit pre-test	Hit post-test	p
17. Microintroduction or MST is defined by the insertion of a larger gauge catheter than the introducer used for its insertion, which makes venous catheterization less traumatic and painful, increasing assertiveness in the first puncture attempt.	5 (71%)	7 (100%)	
18. NB should be preferably positioned in dorsal decubitus and restrained to facilitate catheter progression, without the need for adequate positioning for each insertion site.	5 (71%)	5 (71%)	1* 0.400^†^
19. It is necessary to perform antisepsis of the puncture site with 0.5% alcoholic chlorhexidine for term NB and 0.1% for premature NB, for 30 seconds, and allow the antiseptic to completely dry. The direction of the cleaning movement can be in the “back and forth” direction or with circular movements.	4 (57%)	4 (57%)	1* 0.417^†^
20. After insertion site antisepsis and protection with the sterile field, microintroduction is performed in this sequence: A– Establish access to the venous network through a fine-gauge needle as per the manufacturer; B– After blood reflux, insert the guide wire approximately 5 cm into the needle; C– If necessary, perform the anesthetic button with lidocaine without vasoconstrictor 2 minutes before placing the dilator (peel-away); D– The access needle is subsequently removed over the guide wire, followed by peel-away total placement; E– Insert the PICC catheter until the desired measurement through this dilator. If the catheter progresses successfully, break the peel-away and stabilize the PICC, awaiting confirmation of the distal tip by imaging exam.	1 (14%)	3 (43%)	0.500* 0.364^†^
21. Regarding the performance of local anesthesia by nurses in PICC insertion, the Opinion 15/2014 of the Federal Council of Nursing (COFEN) advises that nurses with training/qualification course for PICC insertion, in an institution that has a protocol that regulates the application of local anesthetic by nurses and professional training for this activity, perform the local anesthesia procedure with 1% or 2% lidocaine without vasoconstrictor in the subcutaneous tissue.	7 (100%)	7 (100%)	1^†^
22. Microscopically, cutting the PICC changes its tip morphology. Therefore, evidence points to fewer complications related to thrombosis when the catheter is trimmed with a guillotine.	7 (100%)	7 (100%)	1^†^
23. The use of adhesive devices for PICC fixation has not been shown to reduce the risk of infection and catheter displacement; therefore, stabilization can be performed using sutures or other materials available at the institution.	5 (71%)	7 (100%)	
24. Any external length of catheter should be stabilized with a slight curve as it exits the skin. This minimizes the risk of accidental device detachment.	7 (100%)	7 (100%)	1^†^
25. When positioned in the subclavian vein, the PICC cannot be considered central. In this vessel, there is an increased risk of thrombosis, infiltration, occlusion and extravasation, complications that can be avoided if the tip of the device is in the cavoatrial junction.	6 (86%)	7 (100%)	
26. If the PICC is inserted in the head, jugular or upper limbs, it is recommended to position the tip between the 3–5 thoracic vertebrae. For punctures performed in lower limbs, it is recommended between 8–10 thoracic vertebrae.	6 (86%)	6 (86%)	1.000* 1.000^†^
27. After the imaging exam, if there is a need to introduce or pull the PICC, there are no contraindications.	4 (57%)	5 (71%)	1.000* 0.087^†^
28. Simultaneous flushing with 0.9% saline, using no resistance for insertion, gently massaging along the vein length, and applying warm compresses to promote vasodilation are considered measures to aid catheter progression.	7 (100%)	7 (100%)	1^†^
29. Slow catheter insertion after blood return impairs PICC progression, due to the risk of clotting inside the introducer, preventing the procedure’s success.	5 (71%)	6 (86%)	1.000* 0.235^†^
30. In case of difficulty in centrally positioning the PICC, it is allowed to perform a more vigorous washing of the catheter with 0.9% saline solution to reposition it in the vena cava.	1 (14%)	2 (28%)	1* 0.588^†^
**Total**	**7 (100%)**	**7 (100%)**	

Source: Survey data, 2021. Note: NUR = applied to nurses. *McNemar’s χ2 test; ^†^Kappa coefficient.

Table 3 shows the number of hits in nurses’ pre- and post-test on maintaining the PICC in MST training.

**Table 3.** Hits in pre- and post-test of nurses participating in the research (n = 7) on peripherally inserted central catheter maintenance in the training of the Modified Seldinger Technique – Campo Largo, PR, Brazil, 2021.

Maintenance	Hit pre-test	Hit pre-test	p
31. It is necessary to assess PICC’s functionality using a 10 ml syringe or a syringe designed specifically to generate low injection pressure, performing an initial flush, slowly aspirating the catheter until blood returns, which is an important component in catheter function assessment, before the administration of drugs and solutions.	7 (100%)	7 (100%)	1^†^
32. The appropriate volume of saline solution for washing the catheter does not need to be followed in care practice, which consists of at least twice the catheter’s total internal volume.	5 (71%)	6 (86%)	1* 0.235^†^
33. The positive pressure technique for PICC flushing allows the reduction of blood backflow into the catheter. Measurement of the circumference of the limb where the PICC is inserted should be done frequently to assess signs of deep vein thrombosis, such as edema.	7 (100%)	7 (100%)	1^†^
34. Disinfect the PICC connections and connectors once every 12 hours with alcohol-based antiseptic solution, with mechanical friction for 5 to 15 seconds.	3 (43%)	5 (71%)	0.625* 0.077^†^
35. In neonatology, changing the dressing that is made of a transparent semipermeable membrane for the PICC should be performed every seven days or in case of detachment/dirt.	4 (57%)	4 (57%)	1* 0.167^†^
36. Healthcare professionals have a duty to document the care provided to patients in relation to infusion therapy (monitoring, patient response, side effects and measures taken).	7 (100%)	7 (100%)	1^†^
37. The need for permanence of central venous access must be reassessed daily by the care team.	7 (100%)	7 (100%)	1^†^
**Total**	**7** **(100%)**	**7** **(100%))**	

Source: survey data, 2021. Note: NUR = applied to nurses. *McNemar’s χ2 test; ^†^Kappa coefficient.

Table 4 presents the comparison between nurses’ assertiveness.

**Table 4.** Comparison between pre- and post-test hits by professional category: nurses (n = 7) – Campo Largo, PR, Brazil, 2021.

Professional category	Moment	N	Mean	Median	Standard deviation	P-value*
**Nurses**	Pre-test	7	5.40	6.00	1.87	0.000
	Post-test	7	5.94	7.00	1.47	

Source: survey data, 2021. Note: *Paired T test.

## DISCUSSION

The MST implementation process through the training of nurses in the NICU showed satisfactory pre-test and post-test reliability. Professionals had prior knowledge in items on device insertion and maintenance, with low assertiveness regarding the indication of technology and procedure for microintroduction via ultrasound on limb repositioning and disinfection of connections/connectors.

The text will be described in the sequence presented in the results and divided into PICC pre-insertion, insertion and maintenance.

### Peripherally Inserted Central Catheter Pre-Insertion

For a new technology to be incorporated into the health system, training and qualification of professionals involved in its implementation is essential. Safe care requires qualified and committed professionals, a favorable environment and their understanding that effective technical work is transformative and requires work organization. In this context, it is important to understand how advanced nursing practices contribute to patient safety, considering the possibility of minimizing risks through differentiated skills and knowledge^(22)^.

With regard to the need for qualification in MST through a specific course, hits predominated in post-test, COFEN Resolution 258/2001 supports PICC insertion by nurses, provided that they undergo qualification through a qualification course^(18)^. As the MST adds different stages to traditional PICC insertion, in addition to being an Advanced Practice in Nursing, new training is required.

The MST technology does not depend on ultrasound use, which is a support in the technique to increase the procedure assertiveness^(13)^, and placement of central venous catheters can be performed without the aid of this imaging device. An integrative review that aimed to identify the scientific evidence about this technology in neonatology showed that the combination of MST with ultrasound is still a recent practice in neonatal units and, therefore, its execution is not always linked to the use of a real-time imaging device^(23)^. Furthermore, ultrasound is not always available in health practice; therefore, radiography is the most used method to confirm the procedure’s success^(24,25)^.

### Peripherally Inserted Central Catheter Insertion

Regarding the appropriation of MST’s sequential stages, it was evident that nurses assimilated the technique with greater clarity after the theoretical-practical training. MST adds some steps in relation to the traditional PICC insertion method. Initially, vessel catheterization is established using a small-gauge needle. After puncture, the flexible tip of the guide wire is inserted from three to five centimeters through the introducer to guarantee access to the venous network. While professionals hold the guide wire in this position, gently withdraw and remove the needle. The next step is to pass the dilator/introducer over the guidewire, inserting it completely through the skin, as it will follow the same path as the guide, i.e., to the center of the blood vessel. Carefully, the guidewire should be withdrawn, leaving only the dilator/introducer in place. At this point, the catheter should be advanced in the vessel to the desired length^(13)^.

Another item that had the greatest hit after the educational intervention was percutaneous catheter stabilization at the moment of PICC insertion. Stabilizing and securing vascular access devices is part of the main infusion therapy guidelines in order to avoid complications and unscheduled removal. Contrary to what was described in question 23, the use of purpose- designed stabilization devices (DSD) contributes to reducing the risk of infection and device displacement. Furthermore, stabilization should not be performed using sutures and/or non-sterile materials. DSD are considered safer than these materials. Non-sterile tape rolls can harbor pathogenic bacteria. Sutures are associated with greater biofilm growth^(20)^.

The Brazilian National Health Regulatory Agency (ANVISA – *Agência Nacional de Vigilância Sanitária*) considers the use of sutureless stabilization devices to reduce the risk of bloodstream infection. It also emphasizes that the fixation of any venous catheter must be performed using aseptic technique; therefore, non-sterile tapes and microporous tapes are contraindicated over the puncture bed^(19)^.

Regarding question 25, which asks about the location of the distal tip of PICC, greater assertiveness was evidenced in post-test. Misplaced catheters have been reported in various anatomical regions, including the arterial system, mediastinum, pleura, pericardium, trachea, esophagus, arachnoid subspace, and at other aberrant sites. However, the ideal tip location of central venous catheters with the highest safety profile is at the cavoatrial junction (CAJ) for any age group^(26)^. Avoid placing the tip in vessels distal to the superior/inferior vena cava, such as the innominate, brachiocephalic, subclavian or iliac veins, as positioning in these veins increases the risk of adverse events^(20)^. For central catheters inserted in the upper extremities, place the distal tip in the lower third of the superior vena cava. For devices inserted in the lower segments, the level of the diaphragm should be considered as an anatomical landmark^(20)^.

Regarding the measures used to facilitate PICC progression within the blood vessel, no statistical significance was observed between the hits in pre- and post-test; however, it is necessary to discuss them, since they are relevant strategies in clinical practice that help professionals on the success of catheter progression. Furthermore, in the case of Brazilian public institutions, the health team does not always have access to recent technologies for the placement of PICCs, leaving only non-invasive maneuvers to direct the tip of the device to the CAJ, in case of difficulty.

In neonates, it is widely recognized that percutaneous catheterization presents a unique set of technical challenges, which are even more potentiated in premature infants, since the smaller the patient, the greater the technical difficulty of the procedure and the anatomical variations^(27)^. Thus, during the practice of PICC insertion, nurses observe, in some cases, device non- progression, which may be associated with stenosis, tortuous veins, venospasm, bifurcations, closed venous valves, thrombosis and hematomas. To overcome the difficulty of progression in both PICC insertion techniques, bolus saline infusion, gentle vein massage in the direction of blood flow, and warm compress to promote vasodilation are suggested^(20,28)^.

Another widely disseminated strategy in the literature is limb repositioning and/or rotation, considered maneuvers that may favor PICC progression during the procedure. A quasi-experimental Brazilian study that described the creation of a maneuver to move the shoulder in NB statistically concluded the inestimable value of this technique, in addition to being easy to apply, without tissue injuries or additional expenses^(28)^.

In addition to the shoulder maneuver, arm and head movements are methods that facilitate PICC advancement and are associated with temporary changes in the positioning of the shoulder and upper limbs, modifying the natural course of the great vessels and facilitating the rectification of probable venous curvatures, promoting catheter advancement to the CAJ^(29)^. The National Association of Neonatal Nurses of the United States also recommends the incorporation of the aforementioned practices to PICC slow insertion inside the vessel to avoid venous irritation and the development of phlebitis. This conduct also allows the catheter to slide more easily in the central circulation with blood flow, requiring a total introduction speed between 30 and 60 seconds. Another complication resulting from rapid catheter introduction into the vein is that this practice can lead to poor positioning^(13)^.

Another non-invasive maneuver that can be used to reposition the PICC tip is the high-flow flush^(26)^, also referred to as the hydrostatic method, which can also be considered flushing, but technically more vigorous, using pressure. In this maneuver, a saline jet force is applied to the catheter in order to straighten the curve in the centerline^(30)^.

This technique used in saline solution allows repositioning of the tip both during PICC placement and post-insertion, however there are some limitations of use. If performed during venous catheterization, it is dependent on ultrasound and real- time tip visualization technology. The potential for device malpositioning is almost guaranteed at some point during its use, as relatively simple changes in intrathoracic pressure, such as sneezing, coughing, vomiting, can contribute to tip movement to an undesirable location^(19,20,26)^.

### Peripherally Inserted Central Catheter Maintenance

Bad positioning of central venous accesses has been described in abundance throughout the literature, and the non- invasive correction of this complication, even in neonates, has been described in the first 24 hours after the diagnosis of malposition^(26)^. Another restriction imposed on using the hydrostatic method is that it should never be performed in catheters that are not power PICC, as such excess pressure in devices that are not manufactured with resistant polyurethane can cause line rupture. Although the power PICC is inserted by MST, this type of catheter is dependent on ultrasound to be inserted. In the present study, we used microintroduction, but through direct puncture in conventional PICC catheters.

Nurses had a higher hit level in post-test referring to the question that discussed the disinfection routine of PICC connections and connectors. The Infusion Nurses Society (2021)^(20)^ recommends that antisepsis should be performed before any drug/solution enters the catheter lumen. This guideline also points out that the main factors that influence the effectiveness of disinfection are the agent used, the time spent and the method of application. In other words, health professionals need to perform the technique by a vigorous mechanical method, with an alcohol-based solution, for 5 to 15 seconds.

The use of 70% isopropyl alcohol is recommended instead of chlorhexidine, since the drying time is 5 and 20 seconds, respectively, making the latter option less favorable in clinical practice^(20)^. Likewise, ANVISA recognizes the disinfection of connections and connectors with alcoholic antiseptic solution, with movements applied in such a way as to generate mechanical friction, from 5 to 15 seconds, as a measure to prevent infection related to health care^(19)^.

The limitations of this study were related to the small number of nurses participating in the research and because it was a study scenario. In general, statistics showed that there was no significant difference between pre- and post-test of these professionals, possibly due to type II error associated with the small number of nurses. Moreover, the lack of national studies on the research object and on the neonatal population made it difficult to compare the results with the Brazilian reality.

As a contribution of this study, the importance of training the nursing team in PICC care is highlighted, since the use of this technology is part of their routine, especially in the NICU. Specialized teams can reduce adverse events by performing safe maintenance on the device. PICC is an innovative technology, increasingly necessary in NICUs, which requires professionals to have technical and scientific knowledge to avoid complications. For this, compliance with good practices related to its use is essential in neonatal care. Another contribution of this research to practice refers to the potential for expansion to other contexts, by using the same process for the incorporation of new health technologies.

## CONCLUSIONS

The MST implementation process in the NICU showed satisfactory pre- and post-test reliability and perfect device insertion and maintenance for nurses. There were more hits in post-test when compared to pre-test. Despite the MST extending some stages of execution over the traditional method of percutaneous catheterization, there was, by nurses, adherence with greater clarity after theoretical-practical training. Training showed that health professionals need continuous and permanent education, especially in the implementation of a new technology.
